# Mixed methods research on satisfaction with basic medical insurance for urban and rural residents in China

**DOI:** 10.1186/s12889-020-09277-1

**Published:** 2020-08-05

**Authors:** Xiaofang Liu, Fang Yang, Wenwei Cheng, Yanyan Wu, Jin Cheng, Weichu Sun, Xiaofang Yan, Mingming Luo, Xiankun Mo, Mi Hu, Qian Lin, Jingcheng Shi

**Affiliations:** 1 Department of Epidemiology and Health Statistics, XiangYa School of Public Health, Central South University, Changsha, 410078 China; 2 The Third Xiangya Hospital of Central South University, Changsha, China; 3 First Clinical Medical College, University of South China, Hengyang, China; 4 Wuhan Hospital of Traditional Chinese Medicine, Wuhan, China; 5 Jiangxi Cancer Hospital, Nanchang, China; 6 Hunan Medical Security Bureau, Changsha, China

**Keywords:** Medical insurance, Satisfaction, Mixed methods research, Importance-performance analysis

## Abstract

**Background:**

There have been few studies on satisfaction with integrated basic medical insurance for urban and rural residents (URRBMI), and satisfaction with URRBMI is not very high because of the complexity of its policies and differences among the insured. The aim of the present study was to explore the factors that influence satisfaction with URRBMI in China and to provide scientific suggestions to the government for how to effectively manage and improve the policy.

**Methods:**

An explanatory sequential design of mixed methods research was used. A quantitative research using a three-stage stratified cluster sampling method was used to randomly select the guardians of pupils who participated in URRBMI (*n* = 1335). The quantitative research was conducted to calculate the latent variables’ scores and path coefficients between latent variables using SmartPLS3.0. With public trust, public satisfaction, and perceived quality as the target variables, important-performance analysis (IPA) was used to explore the important but underperforming factors, which were the key elements to improving satisfaction with URRBMI. A purposeful sampling strategy according to satisfaction level was used to obtain qualitative research subjects from among the quantitative research subjects. A qualitative research was conducted using semi-structured interviews, and the thematic analysis method was used to summarize the interview data.

**Results:**

The three strongest paths were perceived quality to public satisfaction, with a total effect of 0.737 (*t* = 41.270, *P* < 0.001); perceived quality to perceived value, with a total effect of 0.676 (*t* = 31.964, *P* < 0.001); and public satisfaction to public trust, with a total effect of 0.634 (*t* = 31.305, *P* < 0.001). IPA revealed that public satisfaction and perceived quality were key factors for public trust and that perceived quality was of high importance for public satisfaction but had low performance. The policy quality was a determining factor for perceived quality. The qualitative research results showed that the most unsatisfactory aspect for the insured was the policy quality.

**Conclusions:**

This study found that improving quality is key to improving public satisfaction with and public trust in URRBMI. The government should improve the compensation level by broadening the channel of financing for the URRBMI fund, rationally formulating reimbursement standards, and broadening the scope of the drug catalog and the medical treatment projects. The government should establish a stable financing growth mechanism and effective methods of providing health education to improve public satisfaction and public trust.

## Background

In 2016, the State Council issued *The Outline for Healthy China 2030 Plan*, which states that the universal health care system will be further developed via integration with the basic medical insurance system for urban and rural residents in 2030 [1]. Two of China’s three social health insurance schemes, the urban resident-based basic medical insurance scheme (URBMI; launched in 2007) and rural new cooperative medical scheme (NCMS; launched in 2003), were merged to form the urban and rural resident basic medical insurance scheme (URRBMI; launched in 2016) to improve efficiency and equity [2, 3]. URRBMI is a social welfare policy that aims to provide basic medical services or financial compensation for the urban and rural populations (including students and children) who do not participate in the urban employee-based basic medical insurance scheme (UEBMI; launched in 1998). It is a combination of individual accounts and social pooling accounts. Most of the premium is subsidized by the local government, and the rest is paid by the insured.

The quality of public services and the reform of the basic medical insurance system still need to be optimized. The traditional indicators, such as the participation rate, cannot reflect the acceptability of the system to the insured, as medical insurance basically achieves full coverage. Because the insurance is a public service provided by the government, the satisfaction of the insured is an important indicator to assess the current policy implementation of URRBMI and provide a foundation for the government to improve the policy [4].

Studies on satisfaction with the basic medical insurance system have included two levels: theoretical research and practical research [5]. The former focuses on the significance of the satisfaction evaluation and the construction of an evaluation index [6, 7]. The latter is mainly applied in specific administrative areas or research fields to explore satisfaction and the factors that influence it [8, 9]. To date, most research has focused on satisfaction with the NCMS and URBMI, and few studies have focused on the reasons behind the satisfaction with URRBMI [10–13]. Because of the complexity of the policies and differences among the insured, the satisfaction with medical insurance in practice is not high because of the unreliability and inconvenient utilization of policies, inequalities in access, and the moral hazard of service providers [4, 14]. Integrated basic medical insurance has changed the health resource structure and health security system. Analyzing the factors that influence satisfaction with URRBMI will help to optimize the service mechanisms and promote the reform of the medical insurance system [15–17].

To date, most studies on satisfaction with health insurance have involved quantitative research. Previous studies have tested hypotheses, made statistical inferences about causal relationships between influential factors, and provided accurate satisfaction scores. However, quantitative research cannot measure non-quantifiable aspects and cannot provide a thorough understanding of the complexity of the inherent elements of satisfaction. Therefore, it is impossible to understand the dynamic process of the formation of satisfaction [4, 14, 18]. Because satisfaction is a subjective feeling, qualitative research can be used to carry out in-depth explorations and analyses from the perspective of decision-makers to identify hidden problems and to understand the formation and development of satisfaction. However, qualitative research is not suitable for investigating a large-scale population, theoretical assumptions cannot be directly inferred, and the results are only suitable for specific situations and conditions [19–21]. Mixed methods research (MMR) is a methodology that combines quantitative and qualitative research approaches to expand the understanding of research questions and to confirm research hypotheses [22–24]. MMR overcomes the limitations of both quantitative research and qualitative research [25] and allows researchers to comprehensively capture the complexity of the measures as well as to obtain sufficient credibility [26]. Moreover, MMR is essential for generating data for health policy formation in developing countries [27]. Therefore, MMR has received considerable attention in health services research [28, 29].

This study used an MMR design to analyze the factors that affect satisfaction with URRBMI and probe the important but underperforming aspects of URRBMI as well as the reasons for their underperformance.

## Methods

### Research design

**Fig. 1:**
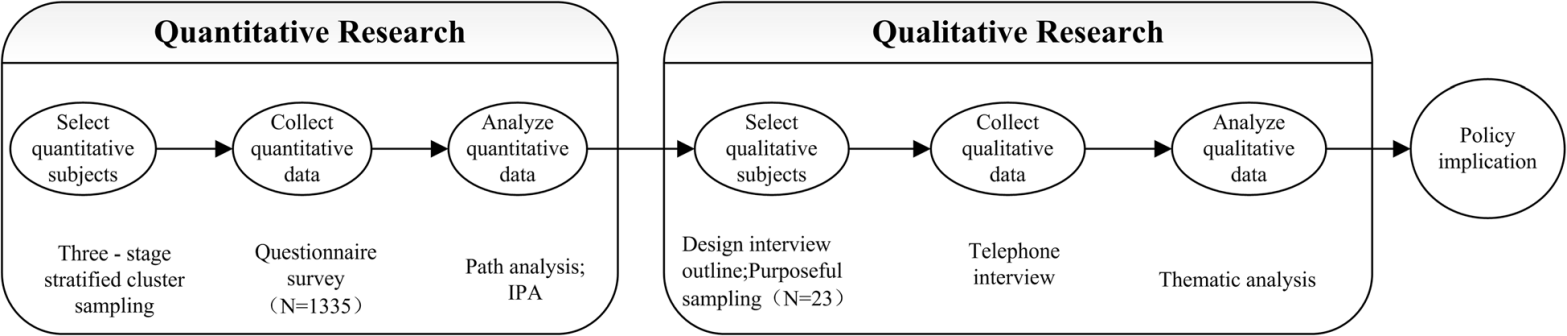
Sequence Interpretation Design Framework

The aim of the quantitative research was to identify insured individuals who were dissatisfied with URRBMI and the factors causing dissatisfaction from the view of insured, while the qualitative research explored the specific details of the factors causing dissatisfaction and analyzed the nature of dissatisfaction from individual experiences. An explanatory sequential MMR design was used [30, 31]. The design is presented in Fig. 1.

Importance-performance analysis (IPA) was used to identify the important but underperforming factors that affect public satisfaction with, public trust in, and perceived quality of URRBMI. Once we identified these factors, we conducted semi-structured interviews with family decision-makers by telephone to identify the factors that caused low satisfaction and the problems with URRBMI to complement the quantitative data. To provide information to guide improving the policy, the findings from the survey and the qualitative interviews were interpreted considering both sources and the synergies across findings.

### The sampling method

The survey was based on schools because this helped with the implementation of the research survey and helped to obtain a good participation rate. Moreover, this approach helped effectively avoid the limitations associated with the elderly who have the medical insurance service but do not understand the specific policy and mobile young individuals in rural areas who do not live at home. Therefore, for the quantitative research, a three-stage stratified cluster sampling method was used to randomly select research subjects from among the most important decision-makers participating in URRBMI in Changsha City. The first stage was to randomly select two districts/counties from nine districts/counties of Changsha City, the second stage was to randomly select two urban schools and two rural schools for each selected district/county, and finally, for each selected school, all students from six randomly selected classes from different grades were chosen. In all, there were 11 latent variables in this study, and based on partial least squares (PLS), the sample should be 10 times the maximum number of latent variables [32], which means the minimum sample size should be 110. Thus, the sample size of 1335 used in this study was sufficiently large.

A purposeful sampling strategy was used to obtain qualitative research subjects from among the quantitative research subjects according to the satisfaction levels of high, medium, and low. Parents who noted they were “willing to interview” on the questionnaire were the potential subjects for telephone interviews. In the qualitative research, the principle behind the sample size was information saturation. The interviewing of subjects was stopped when the content was repeated, and no new topics were presented, indicating that the information was saturated.

### Data collection

The quantitative research was carried out in January 2018. The subjects included in this research were the main decision-makers in each students’ family who were in charge of the purchase and use of the student’s URRBMI. They were responsible for the participation of primary school students in URRBMI, as well as selection of health services and the purchasing of medicines when needed.

Qualitative data were collected by telephone interviews over a three-month period from June to August 2018. The interviewees were interviewed according to a semi-structured interview outline. Oral consent was obtained from each participant upon receiving a full explanation of the purpose and procedure of the study by the researcher. The interviewer told the interviewees that the interview contents would be confidential, how the interview information would be used, and that the interview would be recorded. Informed consent was obtained orally. The interviewer interviewed the interviewees according to the semi-structured interview outline and recorded the entire telephone conversation with the consent of the interviewees. The entire interview lasted about 20–30 min not including repeated interviews.

### Data analysis

#### Descriptive statistics of the respondents

The sociodemographic variables and measurement variables are presented as the mean (SD) for continuous variables that were normally distributed and as the median (QR) for variables with a non-normal distribution. Categorical variables are presented as frequencies (proportions). A two-tailed *P* ≤ 0.05 was considered statistically significant.

#### Path analysis between latent variables

**Fig. 2:**
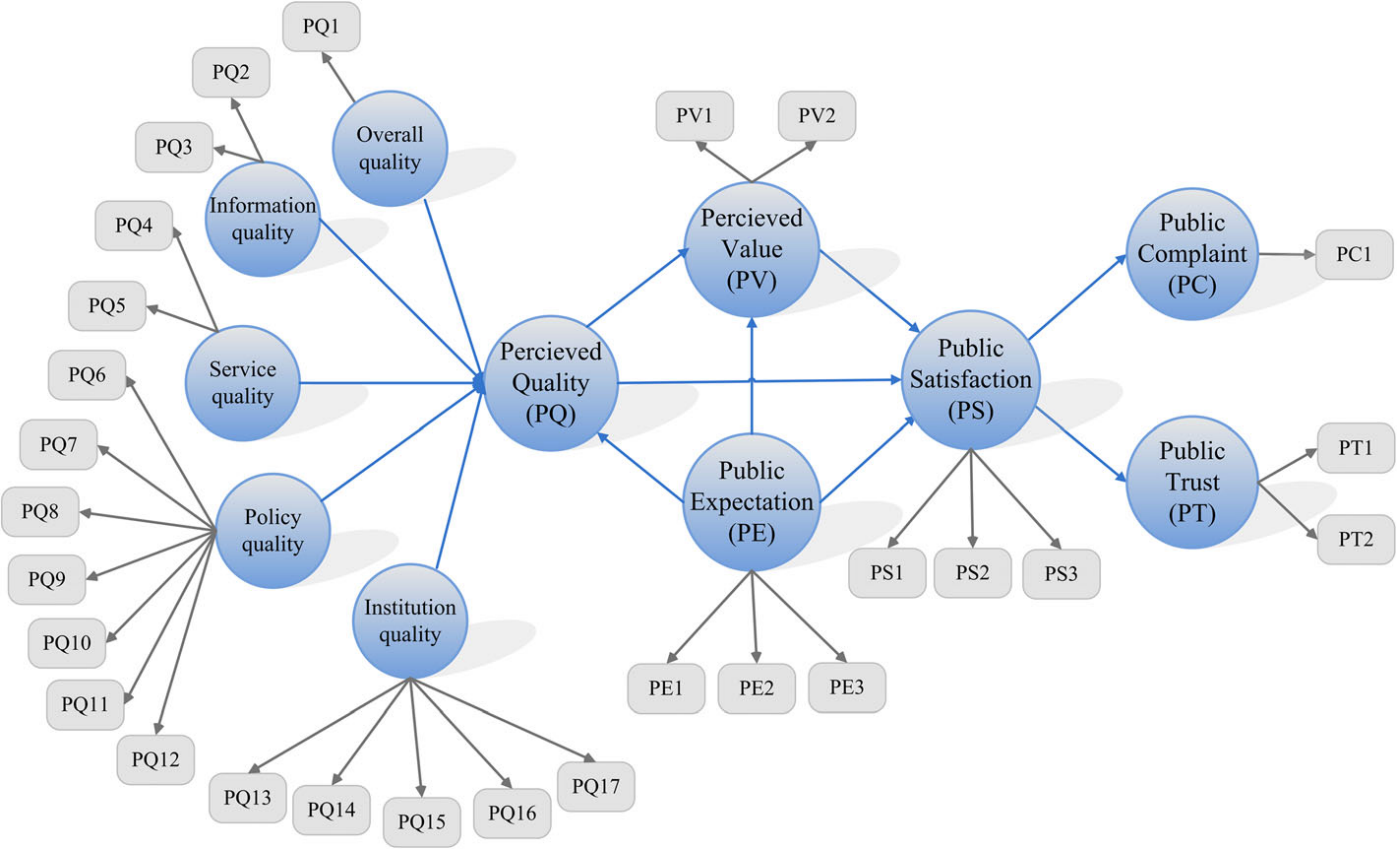
Satisfaction Index Model for Basic Insurance for Urban and Rural Residents

Based on the American customer satisfaction index (ACSI) model, Cheng revised the satisfaction index model for URRBMI (SIM_URRBMI) [33] (Additional file 1). The SIM_URRBMI model is shown below (Fig. 2). The model consists of 11 latent variables, including public expectations (PE), perceived quality (PQ) (which consists of five first-order latent variables, including overall quality, information quality, service quality, policy quality, and institution quality), perceived value (PV), public satisfaction (PS), public complaint (PC), and public trust (PT). Public expectation, perceived quality, and perceived value were the explanatory variables of public satisfaction. Perceived quality was the insured’s evaluation of their recent consumption experience with URRBMI and was expected to have a direct and positive effect on public satisfaction. Perceived value was the perceived level of quality relative to the price paid, which controlled for differences in income and budget constraints across respondents. Public expectation represented both prior experience with URRBMI and a forecast of the insurance service’s ability to deliver insurance service quality in the future. Public complaints and public trust were the outcome variables of public satisfaction. The former encompassed formal or informal complaints about URRBMI from the insured, and the latter was likelihood that the insured would continue to purchase URRBMI in the future.

Compared with a five-point scale and seven-point scale, a ten-point scale reduces the influence of skewness in the distribution data, as satisfaction data typically have a skewed distribution. Therefore, a questionnaire was made by our research team [33, 34]. A 10-point scale (1–10) was adopted for all variables, except for the binary classification (0,1) for public complaint. The SIM_URRMBI model was constructed using a partial least squares structural equation model (PLS-SEM) [35]. SmartPLS3.0 software was used to process the raw data and calculate the scores of the latent variables and the path coefficients between the latent variables with the PLS-SEM.

#### Importance-performance analysis

In 1977, Martilla and James proposed IPA for use in marketing research [36]. IPA is a method of guiding scientific management decision-making by exploring the strengths and weaknesses of service quality in a certain field. It presents the perception of the importance and performance of a policy or service in the form of a four-quadrant matrix.

This method can help the government to identify the strengths and weaknesses of URRBMI in order to enhance public satisfaction and public trust. The SIM_URRBMI model was revised on the basis of the ACSI model, embedding in it a system of cause-and-effect relationships [33]. The chain of causality began with the antecedents affecting public satisfaction, namely public expectation, perceived quality, and perceived value, and ended with their consequences: public complaint and public trust. Public satisfaction was the core variable, located at the center of the casual chain. Based on this framework, we conducted IPA with public satisfaction, public trust, and perceived quality as the target latent variables based on the path coefficients and latent variable scores [37]. The path coefficient of each latent variable in the PLS-SEM analysis on the target latent variable was used as the total effect to judge its importance [38–40], and the score of each latent variable in SIM_URRBMI was used as the performance of each latent variable [37, 41].

The directions of the observed variables of public complaint were changed by reversing the scale, and the scores of all items were converted to a hundred-mark system using formula (1), where Y_ij_ represents the ith data point of the jth latent variable [41, 42].

1 $$ {\mathrm{Y}}_{\mathrm{ij}}=\frac{\left(\mathrm{E}\left[{\mathrm{x}}_{\mathrm{ij}}\right]-\min \left[{\mathrm{x}}_{\mathrm{j}}\right]\right)}{\left(\max \left[{\mathrm{x}}_{\mathrm{j}}\right]-\min \left[{\mathrm{x}}_{\mathrm{j}}\right]\right)}\times 100 $$

**Fig. 3:**
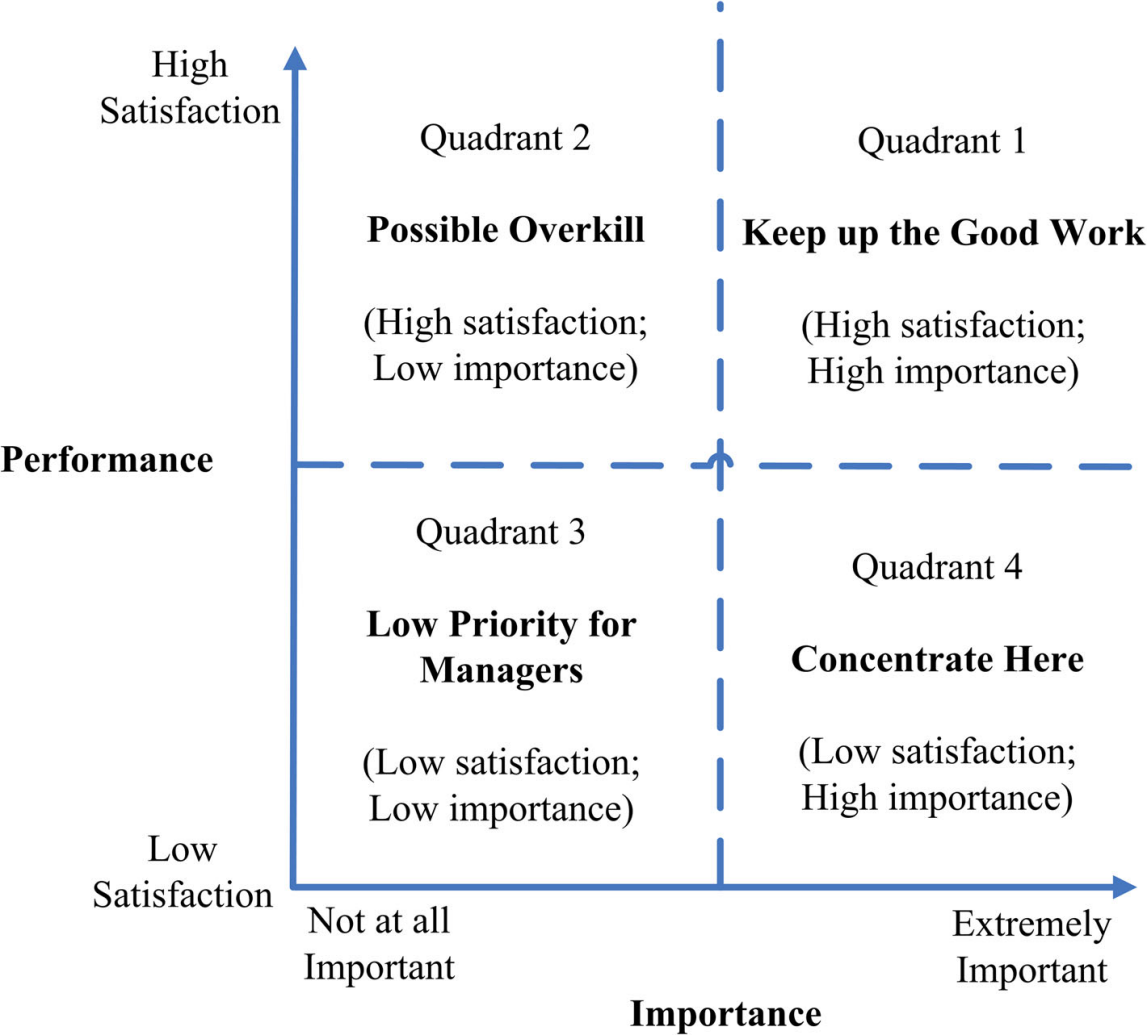
Importance-Performance Analysis grid

The interpretation of the IPA results is graphically presented on a grid divided into four quadrants. Figure 3 presents the IPA grid. The X-axis reports the public’s perceived importance (the total effect of the target latent variables in the SIM_URRBMI model) of the selected attributes, and the Y-axis presents the performance (the score of each evaluated latent variable) in relation to these attributes. The two-dimensional plane is divided into four quadrants based on the average of importance and performance of the corresponding variables [43, 44]. The four identifiable quadrants were: Keep Up the Good Work, Possible Overkill, Low Priority for Managers and Concentrate Here [44].

#### Qualitative data analysis

Using Microsoft Word and Microsoft Excel, at least two investigators coded each transcript together, reconciling discrepancies by discussion until a consensus was reached. The audio recordings of the interviews were transcribed verbatim in Chinese first. Analysis of the interview data following the transcription was inductively performed using thematic analysis of explanatory phenomenology research [25, 45]. The method was divided into six steps: 1) familiarization with the data, 2) generating initial codes, 3) searching for themes, 4) reviewing themes, 5) defining and naming themes, and 6) producing the report. Collection and analysis of the data were continued until the information was saturated.

## Results

### Demographic characteristics of the respondents

A total of 2006 questionnaires were distributed in 8 primary schools and 1807 were recovered (recovery rate was 90.08%). Among these, 1649 questionnaires were valid (effective rate was 91.25%). There were 1335 participants with URRBMI for their children. Table 1 presents the socio-demographic information of the pupils and main decision-makers in the quantitative and qualitative research components. For the quantitative research, the mean age of the pupils was 9.27 years, and the proportions of males (48.68%) and females (51.32%) were similar. The mean age of the main decision-maker was 37.07 years, and most of them were a parent of the pupil (97.07%), predominantly mothers (62.76%). For the qualitative research, the mean age of the pupils was 9.74 years, and the proportions of males (47.83%) and females (52.17%) were similar. The mean age of the main decision-maker was 38.95, and most of them were a parent of the pupil (86.96%), predominantly mothers (65.22%).

**Table 1:** Socio-demographic information of the pupils and main decision-makers

Variables		Quantitative research(***n*** = 1335)	Qualitative research(***n*** = 23)
**Pupils**			
Age, Mean ± SD		9.27 ± 1.74	9.74 ± 1.84
Sex, n(%)	male	646 (48.68%)	11(47.83%)
	female	681 (51.32%)	12(52.17%)
Health status, n(%)	Very good	728 (54.99%)	14(60.87%)
	Good	529 (39.95%)	8(34.78%)
	General	67 (5.06%)	1(4.35%)
**Main decision-maker**			
Age, Mean ± SD		37.07 ± 5.88	38.95 + 7.69
Relationship with pupils, n(%)	Father	457 (34.31%)	5(21.74%)
	Mother	836 (62.76%)	15(65.22%)
	Others	39 (2.93%)	3(13.04%)
Sex, n(%)	male	474 (35.59%)	7(30.43%)
	female	858 (64.41%)	16(69.57%)
Marital status, n(%)	Married	1275 (96.37%)	22(95.66%)
	Divorced	40 (3.03%)	1(4.34%)
	Never Married	4 (0.30%)	–
	Others	4 (0.30%)	–
Education, n(%)	Junior high school and below	372 (28.38%)	6(26.09%)
	High or technical secondary school	583 (44.47%)	13(56.52%)
	Junior college	187 (14.26%)	3(13.04%)
	Bachelor	153 (11.67%)	1(4.35%)
	Master or above	16 (1.22%)	–

### Quantitative research phase

#### Path analysis between latent variables

Table 2 shows that all paths were positive except for four paths for public complaint. The three strongest paths were perceived quality to public satisfaction, with a total effect of 0.737; perceived quality to perceived value, with a total effect of 0.676; and public satisfaction to public trust, with a total effect of 0.634.

**Table 2:** Results of total effect analysis of SIM_URRBMI

Total path	Coefficient	***SE***	***t***	Total path	Coefficient	***SE***	***t***
PE → PQ^a^	0.568	0.022	25.377^*^	PQ → PV^a^	0.676	0.021	31.964^*^
PE → PV	0.588	0.025	23.122^*^	PQ → PS	0.737	0.018	41.270^*^
PE → PS	0.584	0.024	23.953^*^	PQ → PC^b^	− 0.179	0.016	10.870^*^
PE → PC^b^	−0.142	0.013	10.963^*^	PQ → PT^b^	0.467	0.018	25.732^*^
PE → PT^b^	0.371	0.023	16.142^*^	PQ_overal→PQ^a^	0.069	0.002	36.692^*^
PV → PS^a^	0.467	0.032	14.698^*^	PQ_information→PQ^a^	0.110	0.003	31.786^*^
PV → PC^b^	−0.113	0.013	9.010^*^	PQ_service→PQ^a^	0.135	0.003	47.898^*^
PV → PT^b^	0.297	0.023	13.161^*^	PQ_policy→PQ^a^	0.472	0.006	76.267^*^
PS → PC^a^	−0.243	0.020	11.847^*^	PQ_institution→PQ^a^	0.354	0.005	64.894^*^
PS → PT	0.634	0.020	31.305^*^				

^a^ indicates that only the direct path was set, ^b^ indicates that only the indirect path was set, ^*^ indicates *P* < 0.001

#### Importance-performance analysis

The scores of all observed variables in the SIM_URRBMI model are shown in Additional file 2. Based on the score and path coefficient of the latent variables, the importance and performance of each variable were determined, and the results are shown in Table 3.

**Table 3:** Important-Performance Analysis results

Target variables	Latent variables	Importance	Performance
Public trust	Public expectation	0.371	66.608
	Perceived quality	0.467	56.361
	Perceived value	0.297	58.932
	Public satisfaction	0.634	57.753
Public satisfaction	Public expectation	0.584	66.608
	Perceived quality	0.737	56.361
	Perceived value	0.467	58.932
Perceived quality	Overall quality	0.069	67.885
	Information quality	0.110	49.147
	Service quality	0.135	60.476
	Policy quality	0.472	50.256
	Quality of institutions	0.354	62.708

**Fig. 4:**
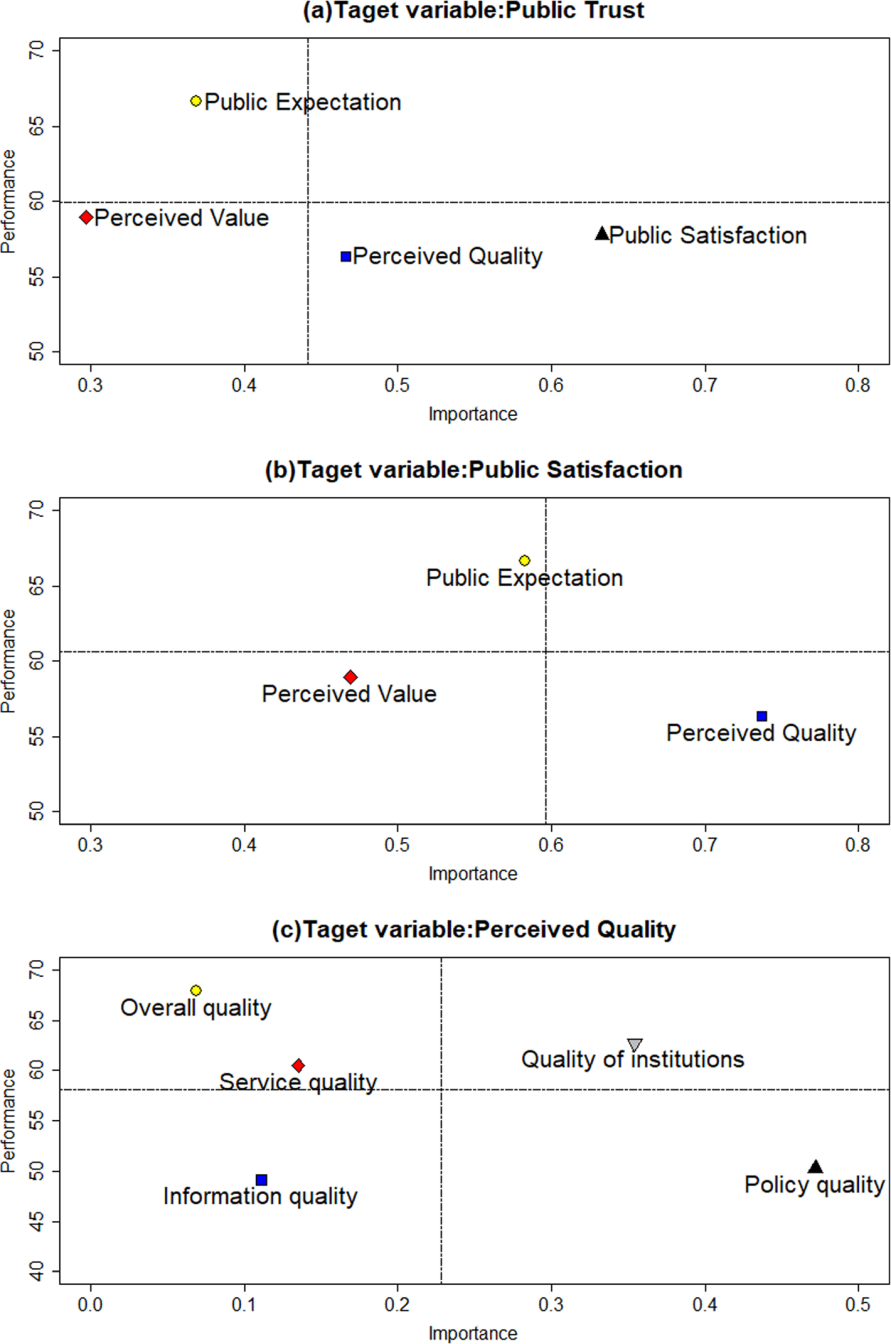
Importance-performance Analysis Results

Public trust was the final variable in the model, and the improvement of public satisfaction was conducive to the improvement of public trust, which would ensure the stability of the insured. As shown in Table 3 and Fig. 4a, the important but underperforming latent variables of public trust were public satisfaction and perceived quality, and the performance of the two variables was only 57.753 points and 56.361 points, respectively. A one-point increase in the performance of public satisfaction and perceived quality increased the public trust performance scores by 0.634 and 0.467, respectively. The latent variable with high performance and low importance was public expectations.

Public satisfaction was located in the center of the model and was the factor that most affected public trust. It was also influenced by perceived quality, perceived value, and public expectation in the model. In Table 3 and Fig. 4b, the important but underperforming latent variable of public satisfaction was perceived quality, and the performance score for perceived quality was relatively low (56.361). In a ceteris paribus situation, the importance of public satisfaction increased by 0.737 with a one-point increase in the performance score for perceived quality.

According to the IPA results of public trust and public satisfaction, the most important variable affecting public trust was public satisfaction, and improving the perceived quality of the insured was the most important and effective measure to increase public satisfaction. Therefore, we conducted IPA with perceived quality as one of the target latent variable. As shown in Table 3 and Fig. 4c, policy quality was the most important factor for perceived quality, and the performance score for policy quality was relatively low (50.256). In a ceteris paribus situation, a one-point increase in the performance score for policy quality increased the performance of perceived quality by 0.472. Service quality and overall quality were high-performing but relatively unimportant aspects for perceived quality and were located in the Possible Overkill area. Institutional quality was located in the *Keep up the Good Work* area and had high importance and performance. Information quality was relatively unimportant, but its performance was low; it was located in the *Low Priority for Managers* area, indicating that it is a potential aspect whose quality needs to be improved.

### Qualitative research phase

**Fig. 5:**
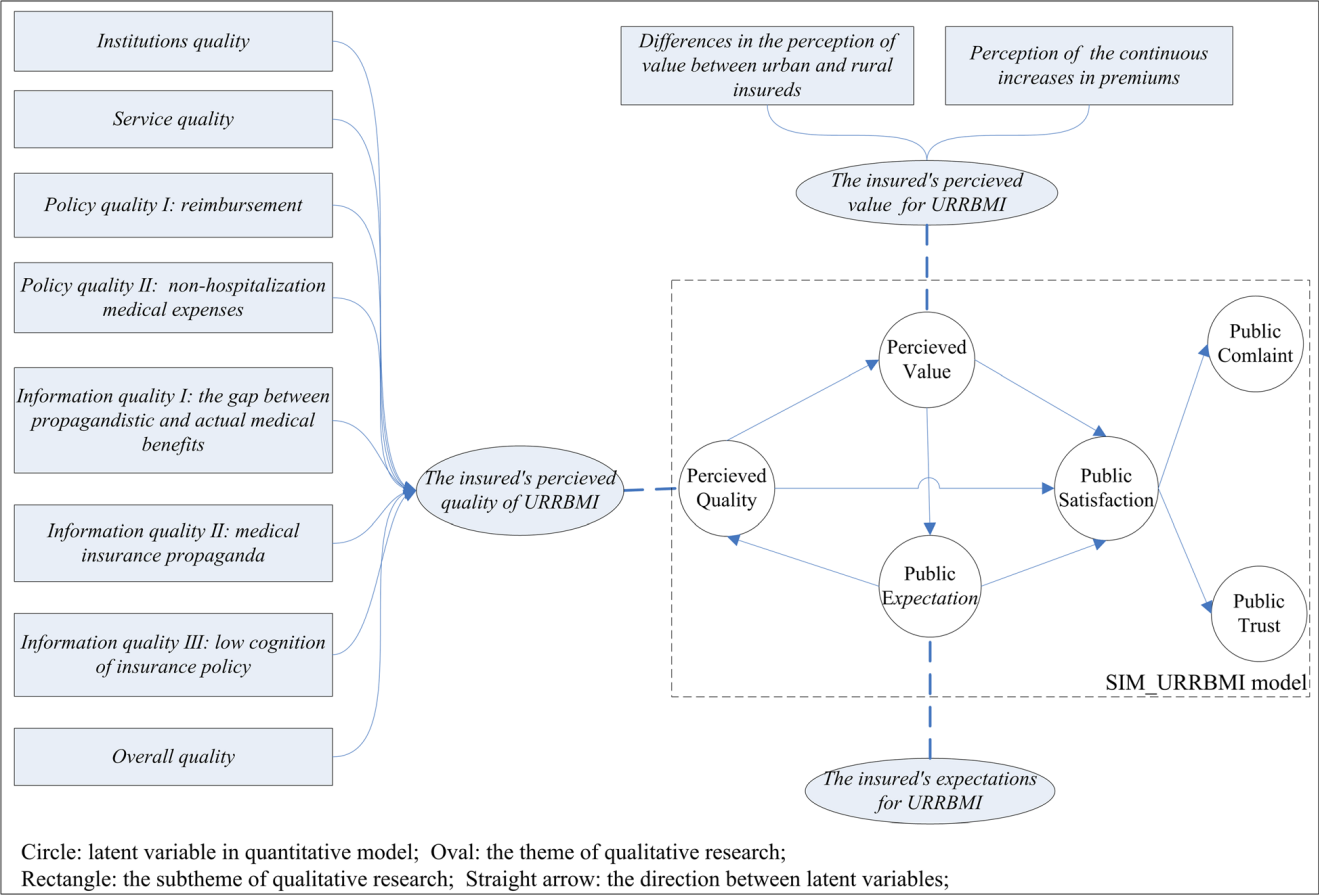
Qualitative Research Theme Map

A total of 23 interviewees were enrolled in the qualitative research phase. Three themes were extracted from the content of the interviews, and Fig. 5 shows the themes derived from our analysis. To promote understanding, we included quotations in the appendices (Additional file 3). This will provide readers with sufficient evidence for evaluating the themes and subthemes we articulated.

#### Theme 1 the insured’ s expectations for URRBMI

Psychologically, the insured believed the notion that enrollment would help them avoid dangers or diseases and ensure their safety. On the other hand, the URRBMI provides fair basic medical security for the insured by compensating them for part of the medical expenses they incur when they experience illnesses or accidents. The insured’s expectation for URRBMI was that basic medical insurance would help share their medical expenses from the treatment of diseases to reduce personal medical expenses and their economic burden.

#### Theme 2 the insured’ s perceived quality of URRBMI

The insured’s views of the quality of URRBMI mainly involved five aspects: 1) institutions quality, 2) service quality, 3) policy quality, 4) information quality, and 5) overall quality.

##### Subtheme 1 institutions quality

The services provided by medical insurance institutions mainly include the procedures for enrollment and reimbursement. Many of the insureds were extremely satisfied with the reimbursement procedures because the health insurance institutions made reimbursement convenient for inpatients and inpatients could be reimbursed after being discharged from any hospital. The insured were satisfied with the procedures for enrollment and reimbursement except for the follow-up work, such as the issuance of vouchers.

##### Subtheme 2 service quality

There are many ways to pay the premium, such as through banks, networks, and full-time staff. Therefore, the insured thought the payment methods were convenient and efficient*.* Although bank payments are more convenient than traditional methods, there are also some problems, such as the long wait time. Compared with bank payments, online payments were more frequently used.

##### Subtheme 3 policy quality I: reimbursement

The intention of URRBMI is to provide the insured with economic security through appropriate reimbursement policies. However, some insured had complained about the current reimbursement policy, including the reimbursement scope, reimbursement rate, deductible, and reimbursement cap line, among other issues.

The main sources of grievance were the reimbursement scope and the reimbursable drug list. The current reimbursement scope did not sufficiently reduce the economic burden of a disease. Some insureds indicated that there were not enough reimbursable drugs, especially drugs for catastrophic diseases. The viewpoint that drugs for catastrophic diseases should be included in the reimbursement scope with an appropriate reimbursement rate was repeatedly mentioned. Some insured did not note the reimbursement rate because the actual reimbursement rate was low and the medical expenses were not adequately reimbursed, which resulted in a financial burden to the insured. Some individuals expressed dissatisfaction with the deductible and reimbursement cap line and held the view that the deductible was too high and that the reimbursement cap line should be reasonably set according to the type of disease.

##### Subtheme 4 policy quality II: non-hospitalization medical expenses

In the view of the insured, non-hospitalization medical expenses refer to medical expenses that cannot be reimbursed by URRBMI, which include the costs incurred in most outpatient clinics, drugstores, and emergency departments. Some patients with chronic illnesses required long-term medication, and their daily medical expenses increased the financial pressure they faced because the medical expenses arising from non-hospitalization treatments could not be reimbursed. In addition, even though they had not been hospitalized after enrolling in the insurance, the daily medical treatments resulted in large medical expenses.

##### Subtheme 5 information quality I: the gap between propagandistic and actual medical benefits

The insured thought there were differences between the benefits that were advertised in the medical insurance policy and the actual benefits, which they learned about during the reimbursement experience. As the insured thought they could not receive all the benefits described in the health insurance policy, they felt that the policy was not well implemented.

##### Subtheme 6 information quality II: medical insurance propaganda

Medical insurance advertising is the foremost way for the insured to obtain medical insurance information, including the frequency, time, content, and method of advertising. The insured indicated that they had a strong desire to obtain insurance information, but they did not receive enough information because of deficiencies in the intensity, content, and method of advertising. The main advertised information was regarding the payment time and premiums, while the information about the benefits was not sufficient to meet the insured’s demands, which made the insured think that the content of the medical insurance information did not make sense. The biggest problem for advertising was the timing because it failed to publicize the insurance policy when the need among the insured was the highest. Daily advertising did not capture the insured’s attention. The greatest need for health insurance policy information is when the insured receive medical services in medical institutions. Therefore, they would be willing to receive this information at that time. Moreover, some of the insured disagreed with traditional advertising methods, such as leaflets and brochures, because these traditional ways were limited to disseminating insurance information that did not meet the insured’s demands for insurance policy information. Instead of the traditional methods, they expected meaningful advertising. For example, young and middle-aged people received daily advertising through the internet, and inpatients wanted to obtain information about the hospital reimbursement policy through the WeChat platform or a paper handbook.

##### Subtheme 7 information quality III: low cognition of the insurance policy

The cognitive understanding of the insured about the health insurance policy was low, especially the specific reimbursement policy. The three main reasons for the low understanding were that the insured had a poor awareness about medical insurance, the majority of family members and the insureds were healthy and the demand for utilizing the medical insurance was low, and medical insurance information was lacking.

##### Subtheme 8 overall quality

The insured had a good overall perception of URRBMI. In particular, the insured had a high opinion of the convenient ways to pay premiums and the rapid and simple reimbursement process. However, the insured still had some poor perceptions of URRBMI, such as the low overall reimbursement ratio, narrow coverage scope for reimbursement, insufficient publicity for medical insurance, simple publicity content, and publicity timing.

#### Theme 3 the insured’s perceived value of URRBMI

The premium is the sum of money that the individuals pay regularly to URRBMI for the insurance policy. Although there were differences in the satisfaction with the premium between urban and rural insureds, they both complained about the continuous increases in the premium.

##### Subtheme 1 differences in theperception of value between urban and rural insureds

There were inconsistencies in the acceptance of premiums between urban and rural insureds. Most of the urban insured stated that the current premiums were acceptable and would not cause financial pressure for their families. However, many rural insured took the opposite view because the whole family participated in URRBMI and the annual premiums did not represent a small amount. There was consistency between the groups in their dissatisfaction with the annual increases in premiums.

##### Subtheme 2 perception of the continuous increase in premiums

Although the urban insured accepted the current premium, some of them complained about the continuous increases in premiums.

## Discussion

### Advantages of mixed method research

Merging the two types of data allowed us to identify areas of concordance and discordance between the qualitative and quantitative results. The advantages of quantitative research are that the path coefficients indicate causal relationships between latent variables in the SIM_URRBMI model, and hypothesis testing can be conducted with statistical inference about the causal relationships. Using qualitative research in this study had the following advantages. First, it increased the rigor of the conclusions, as findings could be checked for consistency. The paths of perceived quality to public satisfaction were positive, and the qualitative results also indicated that the improvement in the quality of URRBMI could meet the demands of the insured and thus increase public satisfaction. Second, it increased the comprehensiveness of the overall findings by showing how the qualitative data provided explanations for the statistical data. The quantitative results showed that the perceived quality was of high importance and low performance, and the qualitative research explained the reasons from the experiences of the insured. Third, the qualitative results provided new ideas for heterogeneity research. The influence of the premium on public satisfaction was not consistent between the urban and rural insured. In the future, subjects can be grouped according to different characteristics to explore whether different groups have different path effects.

### Public trust

Public trust refers to the public having a positive attitude towards the service of URRBMI and being willing to promote it. If the trust of public is satisfied and they also have a continuous preference for URRBMI, then their loyalty to and support of the related government department will be higher. Although the coverage rate of URRBMI is relatively stable, the participation in URRBMI is voluntary, and the premium is paid annually rather than prepaid. Therefore, there will be some discontinuation of insurance because of certain factors such as an increase in the premium, the mobility of the rural population, and the promotion of various commercial insurance products. By improving public trust, the likelihood that the insured will continue to participate and the likelihood that the uninsured will be induced to participate will increase.

The IPA results for public trust revealed that public satisfaction and perceived quality were the key factors that affected the insured’s willingness to continue to participate in URRBMI. Dou’s results also indicated that rural residents’ trust in NCMS was the primary factor affecting enrollment [46]. When the reimbursement demands of the insured were met, the expectation of economic security was realized, and the insured trusted URRBMI. Thus, they will likely continue to participate in the insurance program.

### Public satisfaction

Public satisfaction is the degree of pleasure or disappointment that is felt after receiving medical insurance services compared with the expectations for URRBMI. Kevin’s study showed that satisfaction with URRBMI is quality driven and that an improvement in overall quality will better increase public satisfaction [47]. This was also in line with Fornell’s findings that customer satisfaction was more quality driven than value driven or price driven [35]. The IPA results for public satisfaction revealed that perceived quality was the most important but underperforming predictive latent variable, which was consistent with the results of the study by Kevin [47]. Perceived value was an aspect of low importance and low performance, possibly because URRBMI is a social welfare policy and its premium was much lower than that of other commercial health insurance policies.

### Perceived value

The lower score of perceived value indicated that the insured were less satisfied with the insurance premium. From 2015 to 2018, the premium for URRBMI in Changsha increased from 90 yuan to 180 yuan [48, 49]. The premium increased each year, but the reimbursement benefits did not improve, which caused complaints. After the merging of the two medical insurance systems, the premium for rural and urban participants was same. However, the current premium was much higher than that of NCMS in the past. Unlike families in urban areas, usually all members of a family are insured by URRBMI in rural areas, while their economic sources mainly depend on income from young and middle-aged men. In addition, their income is not steady, especially long-term income, which fluctuates greatly. Thus, the high premiums cause a financial burden. These issues were the reasons for the inconsistent opinions about the premium between the urban and rural insured.

### Perceived quality

Perceived quality was the most critical predictive latent variable for public trust and public satisfaction, suggesting that improving quality should be a high priority in the future. The IPA of perceived quality showed that policy quality was located in the *Concentrate Here* area, indicating that the government should place more focus on policy quality. Interviewees’ dissatisfaction with the health policy was mainly related to the limitations of the reimbursement scope and reimbursable drugs and the low reimbursement rate, which was consistent with the results of previous studies [50]. The insured expected URRBMI to share the financial burden of a disease, but these limitations resulted in a lower actual reimbursement rate. Moreover, the reimbursement deductible was also higher and the reimbursement ratio was lower in high-level medical institutions, which resulted in a lower actual reimbursement rate. However, most of the insured thought that the diagnosis accuracy and treatment level of high-level medical institutions were higher, so they cared about the low reimbursement rate of high-level hospitals. The low actual reimbursement rate was due to the gap in the policy reimbursement rate among different levels of medical institutions and the out-of-pocket items. Therefore, the limitations of the scope of the reimbursement and reimbursable drugs were the main reasons for the lower actual reimbursement rate.

However, URRBMI was designed to provide the insured with basic medical needs. The dissatisfaction was partly due to the imperfect system and partly related to the high expectations of the insured for URRBMI. Some insureds misinterpreted the purpose of URRBMI in that it was designed to provide the insured with basic medical needs. Therefore, they had unreasonable expectations (it was expected that the expenses for some serious or rare diseases, even some operation costs, would be reimbursed) that were not in line with URRBMI’s current reality. On the one hand, this situation reflected that there were deficiencies and drawbacks in policy advertising, which failed to ensure that the insured understood the medical insurance policy correctly. Furthermore, this also provides reference for the development and improvement of the personalized services of URRBMI in the future.

The institutional quality was located in the *Keep up the Good Work* area and was mainly related to the convenient and efficient procedures for enrollment and reimbursement, which should be maintained in the future. The service quality also had a high performance, but its importance was relatively low. The network payment system made it easy to pay premiums, which was the most convenient method for young and middle-aged people who had access to smart phones. However, for older people, there were full-time staff members available who could assist with making the insurance payments through a bank. A variety of insurance payment methods were available to meet different demands. In 2017, Changsha city started the direct reimbursement of hospitalization expenses in different places, and patients only need to pay out-of-pocket expenses when they are discharged from a hospital outside Changsha. The policy shortened the reimbursement process and alleviated the financial burden caused by the advanced payment of medical expenses by the insured in the past [51]. Information quality was located in the *Low Priority for Managers* area, indicating that it was not convenient for the insured to obtain medical insurance information and that advertising for the medical insurance was not sufficient. In addition, the biggest advertising problem for URRBMI was that the timing of policy publicity was not appropriate, and the relevant departments could not be reached when needed. Interviewees were willing to learn about insurance policies only when they needed to use medical insurance for reimbursement. This is consistent with Susan’s finding [52]. There were a few opportunities to obtain an insurance policy when being admitted to a hospital. However, the current publicity on participation is not attracting the attention of the insured. This situation indicated that the relevant departments, such as communities and hospitals, are out of touch. This lack of communication led to the failure to provide adequate policy advocacy services when the insured’s health care demands were the greatest. Therefore, it is critical to promote health insurance to patients who are hospitalized. In addition, there were some shortcomings in health insurance advertising, such as only using a few ways to distribute publicity, an insufficient amount, and its limited content, which affected the insured’s understanding of the insurance policy and their perception of the information quality.

### The influence of policy cognition

An area of discordance was that the insured’s awareness about the medical insurance policy also affected public satisfaction, which was not identified in the quantitative research. Mohammed’s study showed that the greater the awareness of medical insurance policies is, the higher the satisfaction of the insured [16]. Many insureds lacked knowledge about health insurance because they believed that the insurance policy was unified and because they and their families was healthy, which made the insured think that it was not important to learn about and understand the insurance policy. The advertising for the insurance policy failed to provide the insured with relevant policy information and effective access. Reasonable awareness is conducive to improving the enthusiasm of the insured to participate in and utilize URRBMI, as well as their satisfaction and trust in URRBMI.

### Policy recommendations

Therefore, based on these results, the government should take the following steps. First, the government should establish a steady growth mechanism for fundraising. The integration of urban and rural areas in medical insurance is in a transitional period, and the urban economy and rural economy are unbalanced in terms of their development. The setting of the premium should take into account the influence of per capita income, economic growth rate, economic structural differences, and other factors during the transition phase. A steady growth in the premium will help rural residents to adapt to the new basic medical insurance system, which will be conducive to maintaining and improving their satisfaction. Second, the government should increase the payment options for medical insurance funds and rationally formulate reimbursement standards to improve the compensation level. A reasonable reimbursement rate should increase as what is included in the reasonable reimbursement section increases. A reasonable deductible could guide the insured to choose suitable medical institutions. There should be an appropriate cap line for ultra-high medical expenses, such that medical insurance can be preferential used as catastrophic disease insurance. Third, it should improve the scope of the drug catalog and medical treatment projects. With the development of the social economy, the government should bring more drugs, especially necessary drugs for catastrophic diseases, into the reimbursement scope and relax the scope restrictions on outpatient clinics. Fourth, the government should strengthen medical insurance advertising. The advertising methods for URRBMI should be pertinent. For example, for older people, publicity can be promoted through traditional advertising methods, such as brochures. For migrant workers or wage earners, advertising can be carried out in a timely manner using network resources, such as WeChat. It is necessary to establish an effective channel for medical insurance inquiries to make it convenient for the insured to seek help with medical insurance issues. In addition, the insured pay the strongest attention to insurance policy information when they have medical reimbursement demands. Therefore, the relevant institutions can publicize the insurance policy when the insured pay the premium and are hospitalized. There should be an emphasis on special outpatient services that reimburse the cost of outpatient treatment for some chronic non-communicable diseases and catastrophic diseases. Therefore, the government could guide the insured to use URRBMI correctly and effectively through medical insurance advertising.

In summary, the government should consider equal access and benefit to the masses as the starting point and foothold and adhere to tripartite system reform (joint reformation for medical care, medical insurance, and medicine). The government should constantly improve the medical insurance payment system, control the growth rate of medical expenses, and guarantee the allocation of basic drugs to ensure more effective results from efforts aimed at helping the public to obtain medical care and reducing the burden of medical treatments and medications. As a result, government will comprehensively improve the satisfaction with URRBMI and meet peoples’ increasingly growing demands for health care.

### Limitations

URRBMI is a voluntary insurance scheme for rural residents and urban residents who are not covered by basic medical insurance from urban employees (UEBMI), and most of the participants are elderly and children. In rural areas, insureds participate in URRBMI in the form of family collectives, which is different from urban areas because most family members are insured by URRBMI. Therefore, it was not feasible to carry out a community-based survey. The subjects of this study were the main decision-makers in the families of primary school students who were insured by URRBMI; thus, they do not represent all the insured. In addition, there may be differences in the actual satisfaction because some of the subjects had no reimbursement experience. However, the main decision-makers were responsible for the health and lives of the children and the elderly in the family. Moreover, they also tended to be the ones who know the most about health care policies and services. In addition, although the questionnaire asked about the experiences of primary school students with insurance, the main decision-makers would integrate all the family members’ feelings about URRBMI. In addition, the qualitative research also asked about the experiences related to family participation.

This study has some other limitations. First, there was interviewer bias even though interviews are the most common and important method for qualitative data collection. Second, because of the lower education level of a few of the interviewees, there may have been misunderstandings about URRBMI, which prevented them from correctly expressing their views and attitudes. Third, the basic medical insurance uses the district and county as the overall planning unit, and the basic medical insurance policy is slightly different between different regions. The generalization of the research results is limited to Changsha because the samples were selected from Changsha. However, the ideas and results of this study provide a reference for basic medical insurance satisfaction research in other areas.

## Conclusion

This study found that improving quality is key to improving public satisfaction with and public trust in URRBMI. The government should improve the compensation level by broadening the channel of financing for the URRBMI fund, rationally formulating reimbursement standards, and broadening the scope of the drug catalog and the medical treatment projects. The government should establish a stable financing growth mechanism and effective ways of providing health education to improve public satisfaction and public trust.

## Supplementary information

**Additional file 1.** Questionnaire.

**Additional file 2.** Scores Distribution of Observed Variables of SIM_URRBMI Model.

**Additional file 3.** Qualitative Research Findings.

## Data Availability

The datasets used and/or analyzed during the current study are available from the corresponding author on reasonable request.

## Abbreviations

ACSI: American Customer Satisfaction Index
IPA: Importance-performance Analysis
PLS: Partial Least Squares
PLS-SEM: Partial Least Squares Structural Equation Model
SIM_URRBMI: Satisfaction Index Model for Basic Insurance for Urban and Rural Residents
URBMI: Urban Resident Basic Medical Insurance
UEBMI: Urban Employee Basic Medical Insurance
URRBMI: Urban and Rural Resident Basic Medical Insurance
PE: Public Expectation
PQ: Perceived Quality
PV: Perceived Value
PS: Public Satisfaction
PC: Public Complaint
PT: Public Trust
NCMS: New Cooperative Medical Scheme
