# Supplementary material for: Mixed methods research on satisfaction with basic medical insurance for urban and rural residents in China
Source: BMC Public Health. 2020 Aug 5;20:1201. doi: 10.1186/s12889-020-09277-1 (PMC7409480; doi:10.1186/s12889-020-09277-1)
Supplement: Supplementary file 2 — Additional file 2. Scores Distribution of Observed Variables of SIM_URRBMI Model. [file 12889_2020_9277_MOESM2_ESM.docx]

**Supplementary file 2**

**Supplementary Table 1** Scores Distribution of Observed Variables of SIM_URRBMI Model

| **Observed variable** | **$\overline{X}$** | **$S$** | **$X_{min}$** | **$X_{\max}$** |
| --- | --- | --- | --- | --- |
| PQ1 | 7.11 | 2.36 | 1.00 | 10.00 |
| PQ2 | 5.32 | 2.78 | 1.00 | 10.00 |
| PQ3 | 5.51 | 2.65 | 1.00 | 10.00 |
| PQ4 | 6.74 | 2.33 | 1.00 | 10.00 |
| PQ5 | 6.13 | 2.53 | 1.00 | 10.00 |
| PQ6 | 5.86 | 2.33 | 1.00 | 10.00 |
| PQ7 | 5.44 | 2.41 | 1.00 | 10.00 |
| PQ8 | 5.91 | 2.51 | 1.00 | 10.00 |
| PQ9 | 5.47 | 2.48 | 1.00 | 10.00 |
| PQ10 | 5.40 | 2.45 | 1.00 | 10.00 |
| PQ11 | 5.38 | 2.41 | 1.00 | 10.00 |
| PQ12 | 5.25 | 2.39 | 1.00 | 10.00 |
| PQ13 | 6.60 | 2.39 | 1.00 | 10.00 |
| PQ14 | 7.17 | 2.30 | 1.00 | 10.00 |
| PQ15 | 6.54 | 2.13 | 1.00 | 10.00 |
| PQ16 | 6.52 | 2.18 | 1.00 | 10.00 |
| PQ17 | 6.47 | 2.24 | 1.00 | 10.00 |
| PE1 | 6.97 | 2.31 | 1.00 | 10.00 |
| PE2 | 7.11 | 2.20 | 1.00 | 10.00 |
| PE3 | 6.89 | 2.24 | 1.00 | 10.00 |
| PV1 | 6.35 | 2.11 | 1.00 | 10.00 |
| PV2 | 6.26 | 2.10 | 1.00 | 10.00 |
| PS1 | 6.34 | 2.14 | 1.00 | 10.00 |
| PS2 | 6.14 | 2.14 | 1.00 | 10.00 |
| PS3 | 6.09 | 2.16 | 1.00 | 10.00 |
| PC1 | 0.27 | 0.44 | 0.00 | 1.00 |
| PT1 | 6.77 | 2.42 | 1.00 | 10.00 |
| PT2 | 6.70 | 2.51 | 1.00 | 10.00 |
