# Supplementary material for: Mixed methods research on satisfaction with basic medical insurance for urban and rural residents in China
Source: BMC Public Health. 2020 Aug 5;20:1201. doi: 10.1186/s12889-020-09277-1 (PMC7409480; doi:10.1186/s12889-020-09277-1)
Supplement: Supplementary file 3 — Additional file 3. Qualitative Research Findings. [file 12889_2020_9277_MOESM3_ESM.docx]

**Supplementary file 3**

**Supplementary Table 2** Qualitative Research Findings

| Theme | | | Illustrative quotes from enrollee interviews |
| --- | --- | --- | --- |
| Theme 1: The insured’ s expectations for URRBMI | | | *“We don't usually get sick or have accidents, but for those who have an accidental illness, we can make a small contribution.* *The converse is the same.…”*  *“One of my brothers …spent over 300,000 yuan on medical expenses, of which more than 100,000 yuan was reimbursed. That is a great help for a person or a family.”* |
|  |  |  | *“We pay a few hundred yuan a year for the medical insurance, so our family can be safe and family members don’t go to the hospital on account of illnesses or accidents. As for whether I am reimbursed, I don't care. That's what I think.”*  *“The insurance is a kind of prevention. In case of accidents or illnesses, you can be reimbursed for some of the medical expenses.”* |
| Theme 2: The insureds’ perceived quality for URRBMI | | |  |
|  | | Subtheme 1: Institutions quality | *“As long as you bring all the documents that you have prepared, the reimbursement procedures will not be complicated. Thanks to the provincial intranet…I can pay directly in Changsha. I think it is very good, and it is convenient and efficient for the insured.”* |
|  | | Subtheme 2: Service quality | *"Like us, we still have a village accountant who is responsible for the collection of the premium. "*  *“It is also convenient to pay the premium each time, that is, you can go to our local rural commercial bank to pay the premium... this is convenient for us, and the service is very thoughtful.”*  *“I can pay it (the premium) with WeChat since last year. I am very satisfied with this payment method because it is faster. If you go to the bank, you have to queue for one or two hours.”* |
|  | | Subtheme 3: Policy quality Ⅰ: reimbursement | *"My mother-in-law spent over 60,000 yuan when she was hospitalized, but only 10,000 yuan was reimbursed and many expenses could not be reimbursed."*  *"Because there were a lot of hospitalization expenses that weren't reimbursed at the time, such as the cost of surgery. We temporarily hired a doctor to carry out the surgery, but these medical expenses were not reimbursed and a lot of drugs were not reimbursed. I guess that it seems we were reimbursed less than 20,000 yuan. There was not much reimbursement particularly "*  *"My mother got seriously ill with breast cancer this time…there are still some drugs that can't be reimbursed, like some imported drugs. The imported drugs used in chemotherapy are very expensive… not a penny has been reimbursed. I think imported drugs should also be reimbursed, and the reimbursement rate could be lower than that for ordinary drugs, but it should also be reimbursed. "*  *"The medical expenses incurred by our regional hospitals can still be reimbursed, but if we go to hospitals in the urban or higher-level hospitals… there is no reimbursement for medical expenses."*  *"The main reason is that the reimbursement rate is not too high after adding the self-expense items. It would also be acceptable if 70 per cent could be reimbursed. Because the actual reimbursement rate is not too high and includes the threshold fee and out-of-pocket items."*  *"Some hospitals only use drugs that can't be reimbursed... As a result, less reimbursement is made."*  *"If you are in hospital, or a child is hospitalized with a cold, it will cost about 1,000 yuan. Then, that 500 yuan (reimbursement threshold) will not be reimbursed, only about 200 yuan or 300 yuan will be reimbursed, and you will have to spend more than 700 yuan on your own. In fact, the reimbursement rate is only 20% "*  *"I think we should make a distinction between diseases. A catastrophic disease will not be reimbursed if the medical expenses exceed the reimbursement cap. It is really difficult for some families. Because the reimbursement cap is also aimed at people with catastrophic diseases. Common diseases don’t cost too much, but catastrophic diseases…in general, the total cost of treatment is 500,000 or even millions, it should not set the reimbursement cap line "(G3)* |
|  | | Subtheme 4 Policy quality Ⅱ: non-hospitalization medical expenses | *“Daily medication requires long-term medical expenditures. However, the medicine is still not reimbursed.”*  *"When I was in the outpatient clinic,* *the medicines that the doctors prescribed costed hundreds or even thousands of dollars. Why can't these medicines be reimbursed? I don't understand."*  *"Especially when my child went to the dental hospital, the medical expenses were all self-funded. The doctor said that the medical expenses could not be reimbursed and we spent 1,000 yuan. All of the medical expenses were at our own expense! "* |
|  | | Subtheme 5 Information quality Ⅰ: the gap between propagandistic and actual medical benefits | *"I felt that everything in the hospital was different, and it was reimbursed in a different way. "*  *"There is a policy that if the total operation cost and the chemotherapy cost don’t exceed 30,000 yuan and 6500 yuan, respectively, the patient can apply for a medical security for serious illness, for which the reimbursement rate is 60% or 70%. That's much higher than the general reimbursement rate. However, my mom has diabetes… he (the hospital staff) said she was not eligible to apply for that policy because she has an additional disease. "* |
|  | | Subtheme 6 Information quality Ⅱ: medical insurance propaganda | *“I don't know if I didn't hear about it or for any other reason. I don't think our community has done anything about health insurance advertising, maybe I didn't come into contact with it.”*  *"Another reason is that we don't know what kind of protection we can get or what the money will do in the future, all of which doesn't tell us clearly. Anyway, all we know is that you have to pay the premium every year, and we don't know what health care is for. "*  *"How much can I be reimbursed if I go to a different hospital? Some specific policies, such as the benefits available to the insured, which are not clearly stated in the publicity "*  *“Well, there is another way; a leaflet should be provided at the time of admission and medical reimbursement. Because we have to go to work, we seldom notice (health care advertisements) unless we're in the hospital. Sometimes, in the hospital, we will take a serious look. In general, we seldom have time to pay attention to it on weekdays. I guess very few people will pay attention to it (health care advertisements).”*  *“I don’t know because I work out of town. Anyway, I don't know if there are health care advertisements at home, but I don't see this kind of message on my phone.”* |
|  | | Subtheme 7 Information quality Ⅲ: low cognition of the insurance policy | *"Well, I didn't know that, and the hospital never told us about it."*  *"When attending a health care promotion lecture, we don’t listen to that intently. I'm not sick anyway, and I'm not going to use it in the future… As long as it is actually possible to say that someone who needs health insurance, or who has used it, he may listen attentively."* |
|  | | Subtheme 8 Overall quality | *“I don't have any good or bad feeling, I just think it's convenient to enroll in the insurance.”*  *“I think this medical insurance is very good”*  *“Anyway, premium is not too high, and we can reimburse a part of medical treatment expenses”* |
| Theme 3: The insured's perceived value of URRBMI | | |  |
|  | Subtheme 1: Differences in the perception of value between urban and rural insureds | | *“I still feel that the premium is only more than 100 yuan, but it can reimburse so much... I don’t think the premium is expensive, and I still feel that this policy is quite good.”*  *“The premium is still higher for us... We all rely on our daily work to support our family. Every year, our family pays thousands of yuan for medical insurance.”* |
|  | Subtheme 2: Perception of the continuous increase in premiums | | *“The insurance premium was only tens of yuan at the beginning according to our national insurance policy. However, lately, it rose from 100 yuan to 180 yuan.”*  *“If the premiums continue to increase in the next year or the following year, the insured will not accept it.”* |
